# ERRATA

**Published:** 2008-10

Chisholm et al. have reported an error in their article “ Risk of Birth Defects in Australian Communities with High Brominated Disinfection By-product Levels” [Environ Health Perspect 116:1267–1273 (2008)]. In Table 1, the study design, reference, and exposure range given for the first study listed, “Retrospective cohort, Canada,” were incorrect. The results are actually from a cross-sectional study carried out in the United States by Bove et al. (1995), and the exposure range is as follows: High (> 100 μg/L) versus low (< 20 μg/L) THM levels. The defect types and risk estimates (95% confidence intervals) were correct.

The full reference for this study is as follows:

Bove FJ, Fulcomer MC, Klotz JB, Esmart J, Dufficy EM, Savrin JE. 1995. Public drinking water contamination and birth outcomes. Am J Epidemiol 141:850–862.

These errors were introduced during the final drafting stages of the publication; when a much larger table of past literature was reduced, the two studies were accidentally combined. The authors apologize for the errors and emphasize that these changes do not alter the concepts that they addressed in their article.

In the “Conclusion” of the Commentary by Vanderstraeten and Verschaeve [ Environ Health Perspect 116:1131–1135 (2008)], “health,” the last word in the first sentence, should be “exposure.” The corrected sentence is as follows:

Because the overall results from the currently available literature are inconclusive and, in particular, because most of the reported positive findings are flawed by methodologic imperfections or shortcomings, uncertainty still prevails about the possible influence on gene and protein expression from RF exposure at intensities relevant to usual human exposure.

*EHP* regrets the error.

In the article by Zablotska et al. [ Environ Health Perspect 116:1056–1062 (2008)], the units for vitamin A (mg/day) were incorrect in Tables 2–4; the units should be “IU/day.” Also, the units for retinol equivalents in the Appendix should be “μg/day” instead of “mg/day.” The authors regret the errors.

